# Diaphragmatic Dysfunction Following Endoscopic Coronary Artery Bypass Grafting in a Patient With Concomitant Sleep Disordered Breathing: A Case Report

**DOI:** 10.7759/cureus.69978

**Published:** 2024-09-23

**Authors:** Olivier Van Kerkhove, Saartje Demolder, Dries Testelmans, Bertien Buyse, Alexandros Kalkanis

**Affiliations:** 1 Department of Pulmonology, Louvain University Center for Sleep and Wake Disorders (LUCS), University Hospitals Leuven, Leuven, BEL; 2 Laboratory of Respiratory Disease and Thoracic Surgery (BREATHE), Katholieke Universiteit (KU) Leuven, Leuven, BEL

**Keywords:** diaphragmatic dysfunction, endoscopic coronary artery bypass grafting, inspiratory muscle training, lung ultrasound, obstructive sleep apnoea

## Abstract

Unilateral diaphragmatic paralysis (UDP) is a frequent complication following cardiac surgery, usually affecting the left hemidiaphragm. Here, we present a case of a right-sided UDP following endoscopic coronary artery bypass grafting (CABG), which is far more uncommon. A 60-year-old male patient presented at our outpatient clinic with exertional dyspnoea and orthopnoea. Breathing sounds were diminished upon auscultation of the right lung base. He recently underwent a CABG through video-assisted thoracoscopic surgery (VATS). We documented a new right-sided UDP as well as severe obstructive sleep apnoea syndrome (OSAS) in this patient. We started inspiratory muscle training for the diaphragm palsy as well as continuous positive airway pressure (CPAP) therapy to ameliorate his OSAS since this is negatively influenced by UDP. The combination of this particular surgical method, the concomitant OSAS, and the right side of the UDP make this a unique case. In this report, we will briefly summarize several aspects of diaphragmatic dysfunction in the post-CABG setting, with a thorough focus on the role of ultrasound in its diagnosis and follow-up.

## Introduction

Unilateral diaphragmatic paralysis (UDP) is a frequent complication following cardiac surgery and has been reported in 30-75% of patients following coronary artery bypass grafting (CABG) [1,2]. Most commonly, post-CABG diaphragm dysfunction occurs on the left side due to the usage of cold slush to cool the left ventricle [3]. We present an uncommon case of right-sided diaphragmatic paralysis following an endoscopic CABG, complicated by the simultaneous presence of severe obstructive sleep apnoea syndrome (OSAS).

This article was previously posted to the Research Square preprint server on April 10, 2024.

## Case presentation

A 60-year-old retired male patient, a former smoker (40 pack years), presented at the ambulatory pulmonary clinic with progressively worsening dyspnoea on exertion. His medical history revealed multiple percutaneous coronary interventions due to extensive coronary disease, heart failure with a mildly reduced ejection fraction, and type 2 diabetes. Five months ago, he underwent an endoscopic CABG for triple vessel coronary disease. During the video-assisted thoracic surgery (VATS), the right internal mammary artery (RIMA) was completely harvested and used as a free Y-graft on the left internal mammary artery (LIMA), which was only partially dissected. The LIMA was grafted on a diagonal branch of the left anterior descending artery (LAD). The RIMA was grafted on a side branch of the circumflex artery and on the right descending posterior artery. The procedure was performed on-pump with the help of cardioplegia (cold slush). A left-sided haemothorax developed on the first post-operative day and was drained during a new VATS procedure.

The patient experienced dyspnoea on minimal exertion and orthopnoea immediately after his cardiac surgery, which did not resolve after the drainage of his haemothorax. No clinical improvement was seen despite four weeks of intense cardiac rehabilitation. He mentioned no coughing, mucous production, or fever. During the physical examination, the patient was breathing calmly, and his oxygen saturation in ambient air was within normal values.

**Figure 1:**
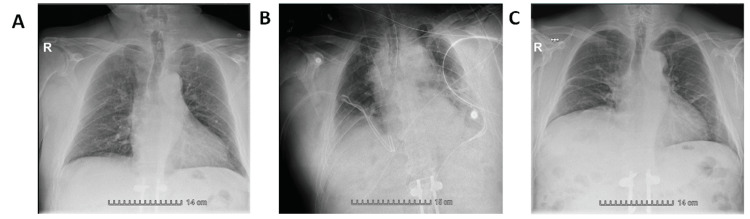
Serial radiographs. Panel A was taken pre-operative, showing normal bilateral diaphragm domes. The RX taken in panel B was taken day 1 postoperative, showing a consolidation of the left lower lobe. The diaphragm is hard to evaluate because of the supine position and positive pressure ventilation. Panel C shows the RX taken a few week after surgery, showing an elevation of the right diaphragm dome.

**Figure 2:**
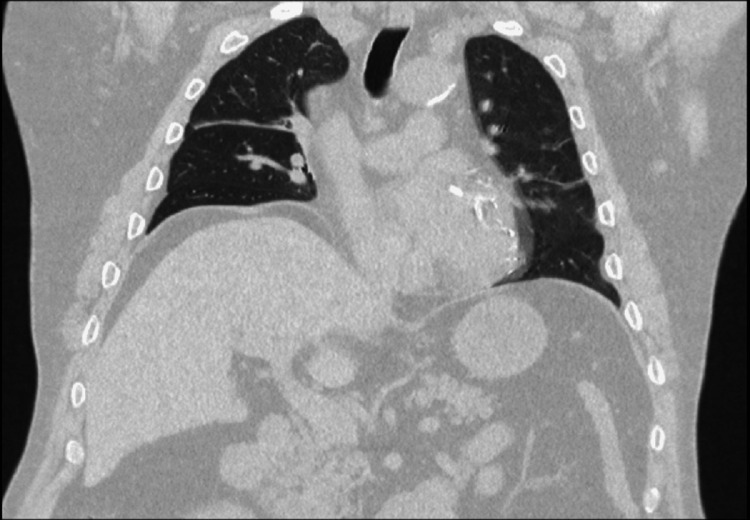
Coronal sections of chest CT scan. There are no structural lesions of the lung and liver parenchyma.

Breathing sounds were diminished at the right lung base. There were no clinical signs of heart failure. Pulmonary function test (PFT) showed a restrictive pattern (FVC 74% pred. - FEV1/FVC 77% - RV 55% pred. - TLC 64% pred.) and a slightly decreased diffusion capacity (76% pred.). Serial radiographs are shown in Figure 1, demonstrating the onset of an elevated hemidiaphragm on the right side immediately after his cardiac surgery. A chest CT scan was performed that confirmed the right-sided diaphragmatic elevation with limited concurrent atelectasis (Figure 2). Chest CT revealed no underlying pathologies. Second spirometry in the upright and supine positions showed a significant decline in FVC in the supine position (24%). Arterial blood gas showed no evidence of daytime hypoventilation (pH: 7.4; pO_2_: 77 mmHg; pCO_2_: 36 mmHg).

**Figure 3:**
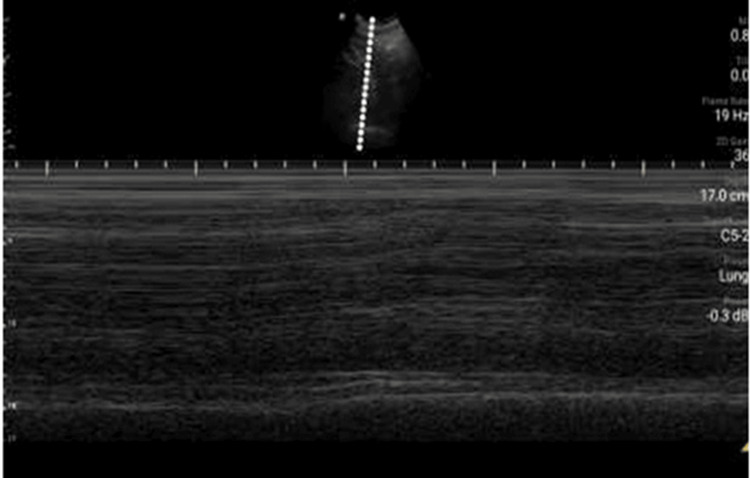
Subcostal echographic view of the right hemidiaphragm on M-mode. A severely diminished excursion could be seen during inspiration.

Further work-up included a phrenic nerve stimulus-detection procedure, showing a partial loss of conductance (latency time was 12 msec on the right side compared to 7 msec on the left). Diaphragmatic ultrasound was carried out next via the subcostal view and showed a severely diminished excursion during deep inspiration of 1.7 cm (Figure 3). Biochemical analysis showed a normal function of the thyroid gland (0.86 mU/L) as well as normal creatine kinase levels (<300 U/L). The diagnosis of a right-sided UDP following cardiac surgery was made.

**Figure 4:**
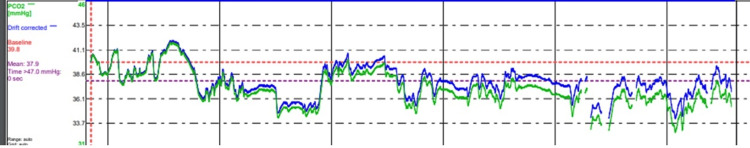
Nocturnal capnometry during polysomnography. Maximum pCO_2_ (blue line) is 42 mmHg, showing no evidence for nocturnal hypercapnia.

As a part of an extensive workup, our patient was screened for co-existing conditions. In-depth anamnesis revealed morning headaches and chronic snoring. Thus, a hospital polysomnography supplemented with a capnography was performed. There was no evidence of nocturnal hypoventilation (Figure 4), but a severe OSA (apnoea/hypopnoea index: 41/h) with frequent desaturations (minimal saturation: 84%; oxygen desaturation index: 36/h; saturation below 90% during 5.4% of the recorded time) was documented. Continuous positive airway pressure (CPAP) therapy was started (pressure: 7.8 mbar), with good compliance and improvement of his complaints after one month of treatment. Simultaneously, the patient was put on a rehabilitation program focussing on the inspiratory muscles, with subsequent clinical improvement. His initial follow-up after the first three months of CPAP treatment was uneventful, and further ultrasonographic follow-up along with his routine CPAP treatment follow-up was scheduled.

## Discussion

We describe a rare presentation of a known side-effect of cardiac surgery, complicated with concomitant OSAS. The specific surgical method used, the ultrasonographic evaluation, and the presence of sleep apnoea make the case stand out from the existing literature and provide us further insight into the pathophysiology and an up-to-date and holistic approach to a complex case.

As mentioned above, dysfunction of the hemidiaphragm is a frequent complication following cardiac surgery. Topical cooling of the left ventricle is the culprit in most cases, which explains why left-sided dysfunction is much more common than right-sided dysfunction [3]. Harvesting of the RIMA can be a possible cause of right-sided diaphragm dysfunction. In an observational study, the incidence of diaphragm dysfunction following RIMA harvesting was about 4% [4]. The right phrenic nerve is in closer proximity to the RIMA and is thus susceptible to injury during this procedure. Dissection of the pericardiophrenic artery ends the blood supply to the phrenic nerve, causing infarction and paralysis. Central venous cannulation is another cause of right phrenic palsy, which has been described in previous case reports [5,6].

The diagnosis of diaphragmatic paralysis is based on confirming both reduced diaphragm function and identifying an underlying cause when present [7]. PFTs and imaging are crucial in establishing the diagnosis. Diaphragm dysfunction can be visualized with all known imaging modalities, but ultrasonography is of particular interest because it is easy to apply at the bedside, can be used in follow-up, and has no side effects. Several parameters are obtained and assessed: diaphragmatic thickness, thickening fraction during inspiration, diaphragmatic excursion, and the velocity of contraction. The cut-off values for these parameters depend, among others, on gender and type of breathing (quiet breathing versus ‘sniff’), but when combined, they provide a good indication of the presence of a diaphragm dysfunction [8]. In our case, only a minimal excursion of the diaphragm could be seen, indicative of a clearly reduced muscle function.

An additional comorbidity that is present in our case is the concomitant OSA. While the sleep-related symptoms were present before the operation, we cannot be certain about the effect of the two pathologies on each other. A recent review studied the impact of UDP on sleep-disordered breathing (SDB) and demonstrated the possible association between UDP and increasing SDB severity, particularly during rapid eye movement (REM) sleep [9]. In the reviewed literature, supine position and REM sleep were associated with obstructive and mixed (both obstructive and central) events, respectively. Compared with controls, UDP was associated with a lower mean and minimum oxygen saturation as well as arterial oxygen tension during all sleep stages and in all body positions. A paradoxical upward movement of the affected hemidiaphragm leading to smaller lung volume and loss of traction may increase airway collapsibility. This sequence of events has been put forward as a potential pathophysiological mechanism. The smaller lung volume caused by this upward movement can deteriorate ventilation, especially during REM sleep (when skeletal muscles are relaxed) and when lying supine [9].

Most patients experience a complete recovery of the phrenic nerve function over time [10]. However, a significant proportion of patients have persisting and symptomatic phrenic palsy. Inspiratory muscle training is a valuable treatment option in these patients, as improved functional capacity has been shown in randomized controlled trials [11,12]. Ultrasonography has been successfully used in the follow-up of diaphragm dysfunction, showing a good correlation with functional capacity [13].

A more radical treatment option is the surgical plication of the diseased hemidiaphragm. This option is reserved for selected patients with persisting paralysis and symptoms despite inspiratory muscle training. However, two case reports have already been published about early surgical plication for patients with post-cardiac surgery diaphragm dysfunction [14,15]. However, we recommend a more conservative approach, as spontaneous recuperation can be seen in the majority of patients [10].

In our case, the lack of clinical improvement after one month of intense cardiac rehabilitation led to a complete diagnostic work-up and the diagnosis of UDP. The existing literature demonstrated that several pathophysiologic paths could have been crossed, and a complete and holistic approach was needed.

First of all, cardiac surgery needs to be taken into consideration as a potential cause of a right-sided UDP, even when a thoracoscopic approach is used [16]. The following key point is that concomitant respiratory disorders should always be held in mind in clinically challenging cases of UDP since the latter has a negative influence on these prior existing diseases. In our case, UDP could further negatively influence nighttime ventilation, and the underlying untreated OSA could contribute to the clinical presentation. Treatment of the pre-existing respiratory disease is a primordial part of the holistic approach.

## Conclusions

To the best of our knowledge, we presented the first case of right-sided diaphragmatic dysfunction following an endoscopic CABG. While left-sided phrenic palsy is commonly seen after cardiac surgery, its right-sided counterpart is much more uncommon. UDP following an endoscopic CABG has not yet been published in the literature, which makes this a unique case. Different mechanisms can account for phrenic nerve damage. Echography of the diaphragm is a useful tool for the diagnosis and follow-up of diaphragmatic complications after cardiac surgery. It is important to screen for respiratory comorbidities, as these are negatively influenced by UDP.
